# Efficacy of transdermal flunixin in mitigating castration pain in piglets

**DOI:** 10.3389/fpain.2022.1056492

**Published:** 2022-11-11

**Authors:** Magdiel Lopez-Soriano, Victoria Rocha Merenda, Pedro Henrique Esteves Trindade, Stelio Pacca Loureiro Luna, Monique Danielle Pairis-Garcia

**Affiliations:** ^1^ Department of Population Health and Pathobiology, College of Veterinary Medicine, North Carolina State University, Raleigh, NC, United States; ^2^ Department of Veterinary Surgery and Anesthesiology, School of Veterinary Medicine and Animal Science, São Paulo State University (Unesp), Botucatu, Brazil

**Keywords:** pain, castration, transdermal flunixin, swine, pain scale, UPAPS, animal welfare

## Abstract

Castration is a painful procedure performed in swine and to date, there are no approved products available in the US to alleviate this pain. Previous work evaluating the efficacy of flunixin meglumine has shown promise in mitigating pain in swine, but no work to date has evaluated transdermal flunixin efficacy in mitigating castration pain in piglets. Therefore, the objective of this study was to evaluate the efficacy of transdermal flunixin (TDF) in mitigating castration pain utilizing a previously validated behavioral pain scale. A total of 98 Large White x Duroc cross male piglets from 98 litters were enrolled in this study. Piglets were randomly assigned to the following treatments: (1) TDF plus castration (3.33 mg/kg; CF; *n* = 24), (2) TDF plus sham castration (3.33 mg/kg; SF; *n* = 26), (3) topical physiological saline plus sham castration (S; *n* = 24), or (4) topical physiological saline plus castration (C; *n* = 24). All treatments were administered 24 h prior to castration. Four-min continuous videos clips were collected 24 h before castration (−24 h), immediately post-castration (0 h), and 24 h post-castration (+24 h). Video clips were then observed and scored by one trained observer using a 4-point pain scale (score 0–3) encompassing the five behavioral domains of the pig acute pain scale (UPAPS). Total pain score averages were analyzed as repeated measures by analysis of variance applying a multilevel model. The UPAPS effectively distinguished varying levels of painful and non-painful states in castrated piglets as observed *via* deviations in total pain scores across timepoints (*P* < 0.0001), treatment (*P* < 0.001) and treatment*timepoint (*P* < 0.0001). Immediately post-castration (0 h), piglets in the C and CF group demonstrated greater total average pain scores than piglets in the S (*P* < 0.03) and SF (*P* < 0.01) groups and castrated piglets treated with TDF demonstrated lower total pain scores (*P* < 0.05) and required less analgesic intervention immediately post-castration compared to castrated piglets receiving no treatment (*P* < 0.0001). For C group 54% required rescue analgesia compared to 29%, 8% and 0% for CF, SF and S piglets respectively. Future work should evaluate implementation of this pain management protocol on a wide scale commercial farm setting.

## Introduction

Societal concerns have increased regarding the health (1), welfare (2), and the conditions of commercially reared swine and therefore should be protected and improved (3). These concerns have increased over the past decade with particular criticism focusing on the lack of pain management protocols implemented with husbandry practices such as castration (2, 4, 5). Castration is commonly performed on US swine farms and in many other countries to improve meat quality by reducing androsterone and skatole concentrations (6), both of which are responsible for the production of boar taint. Boar taint is an unpleasant odor or taste in the meat that can be detected by consumers during the process of cooking or eating pork products. Boar taint presence may not only result in a negative eating experience by consumers but can have significant economic impacts to producers with the potential to reduce profitability as much $27.7/100 kg (hot carcass weight 7).

Nevertheless, castration is a painful procedure (8, 9). It has the potential to negatively impact pig welfare and productivity when performed without anesthesia and analgesia (10) and can increase morbidity and mortality rates among pre-weaned male piglets (8, 11, 12). Currently, there are no pharmaceutical products approved by the US Food and Drug Administration (FDA) to attenuate pain in swine (2, 13). This is in contrast to other swine producing countries in Europe, that currently have over 20 products specifically approved for pain mitigation in swine.

In order for the US to obtain governmental approval for products to be used for pain relief in swine, pharmaceutical companies must first demonstrate product efficacy based on clinically validated biomarkers and tools. Species-specific validated pain assessment instruments are essential to recognize and quantify pain (14, 15). Pain assessment scales have been validated in a variety of species (16, 17, 18, 19) and recently, an acute pain scale for pre-weaned piglets (UNESP-UPAPS) was validated in the US (Robles et al., submitted). The validation of this scale was a critical step in moving the US swine industry forward by providing an objective behavioral tool to quantify pain and assess pharmaceutical product efficacy.

Flunixin meglumine is a non-steroidal anti-inflammatory drug (NSAID) that inhibits cyclooxygenase production and suppresses prostaglandin synthesis (20). Banamine® Transdermal (flunixin transdermal solution, Merck Animal Health. Madison, NJ, USA) is the only drug currently approved for pain control in livestock species and is specifically labeled for mitigating pain associated with foot rot in cattle (21). Previous work evaluating the efficacy of flunixin meglumine has shown promise in mitigating pain associated with lameness in swine (22), tail docking in lambs (23), and castration in lambs (23), goats (24) and calves (25). In addition to the efficacy of flunixin on mitigating castration pain across species, transdermal flunixin has the potential to serve as one of the most realistic pain management options to implement on large-scale commercial swine systems given the ease of applicability and long half-life. Therefore, the objective of this study was to evaluate the efficacy of transdermal flunixin (TDF) in mitigating castration pain utilizing a previously validated piglet acute pain scale.

## Materials and methods

This study was approved by the Institutional Animal Care and Use Committee of North Carolina State University (IACUC protocol 20-113-01) and animals were cared for and handled in accordance with the Guide for the Care and Use of Agricultural Animals in Research and Teaching (26). This study was part of a larger study that was conducted by Merenda et al., (2022) from January to March 2021 at a commercial swine breeding facility located in the Southeastern United States.

### Animals, housing, and management

A total of 98 Large White x Duroc cross male piglets from 98 litters (2–8 days of age), were enrolled in this study. Only one piglet per litter was enrolled in the study and treatment was only applied on the litter level. Piglets were housed with sows on fully slatted, tunnel ventilated farrowing rooms. Room temperature was managed through a computerized control system at 22° ± 1.0 °C. Within each room, sows and litters were housed in individual farrowing crates (2.5 m × 0.7 m) with additional space for piglets (2.5 m × 1.3 m) surrounding the crates. To avoid cold stress on piglets, a heat mat was set to approximately 30–35 °C and lighting was turned on between 600 h and 1630 h.

### Study design and treatments

Piglets were enrolled in the study if they met the criteria described in Table 1 (adapted from 28). Piglets were ear tagged 24 h before castration was performed (Allflex Global Piglet ear tags, Allflex Livestock Intelligence, Madison, WI) to track them down during the video scoring. Piglets were randomly assigned to one of four treatments: (1) transdermal flunixin (TDF) applied topically followed by surgical castration (3.33 mg/kg; CF; *n* = 24), (2) TDF applied topically followed by sham castration (3.33 mg/kg; SF; *n* = 26), (3) physiological saline followed by sham castration (S; *n* = 24), or (4) physiological saline followed by surgical castration (C; *n* = 24).

**Table 1:** Inclusion and exclusion criteria utilized for piglets at the time of enrollment.

Inclusion criteria	Exclusion criteria
Two to five days of age^a^	Clinical signs of disease
Full tails intact	Treatment with any type of antibiotic^b^
Both testicles descended	
Body weight greater than 0.5 kg Maintained within litters with at least five additional male siblings	

^a^ Cross-fostering was permitted prior to enrollment in the study.

^b^ Sows nursing the litter that received any type of antibiotic were excluded from enrollment.

### Drug administration

Treatments were applied 24 h prior to castration as previously described (27). Transdermal flunixin (3.33 mg/kg) and topical physiological saline were administered as an equivalent volume (0.1–0.3 ml/ piglet) and applied topically along the dorsal midline next to the tail head using a disposable syringe (BD disposable syringes, Fisher Scientific, Waltham, Massachusetts) by one researcher. Due to the minimal amount applied to the piglet, it is unlikely that the unabsorbed drug could have been transferred to other littermates. To minimize bias for the observer watching the video, cornstarch (Great Value®, Walmart, Bentonville, AR) and pink non-toxic dye (Pink Rose®, LorAnn Oils, Lansing, Michigan) was added to the physiological saline to mimic the color and consistency of the TDF. This was the first study that applied transdermal flunixin within a litter and not an individually housed pig. Transfer of drug was a possibility; therefore, treatment was applied at the litter level to eliminate potential confounding.

### Castration procedure

Castration was performed by one trained caretaker. Piglets were picked up, individually held, placed in dorsal recumbency, and two vertical incisions were made using a scalpel blade. Once the incisions were made, testicles were exposed, spermatic cords cut, and testicles were completely removed by traction. A sham castration was performed to mimic similar handling conditions in which piglets were picked up, individually held, placed in dorsal recumbency, and had pressure applied to the scrotal area by the same individual castrating.

### Video recording for behavioral assessment

**Figure 1:**
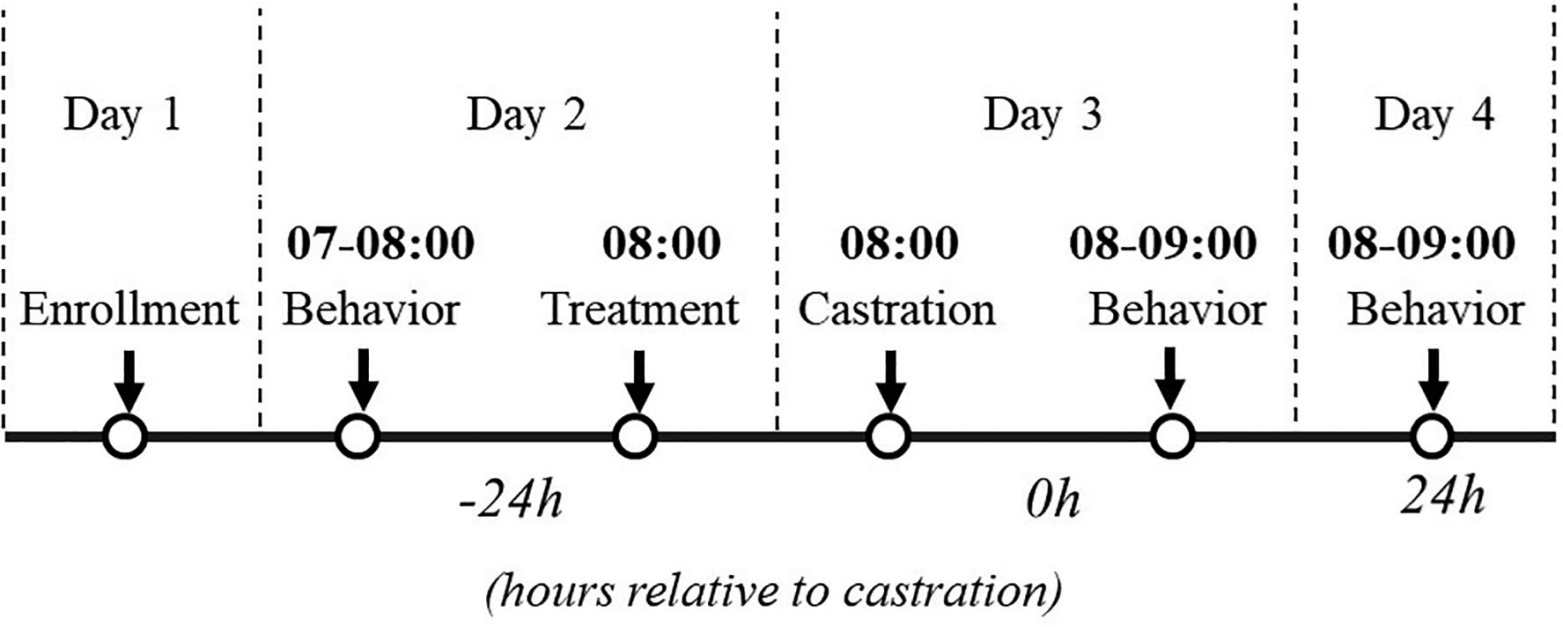
Study design and data collection. Treatment: physiological saline or transdermal flunixin. Castration: surgical castration or sham castration. Behavior: video recording continuously for one hour.

High-definition cameras (Sony HDR-CX405; New York, NY, USA) were installed to the back of the farrowing crate using zip ties with the camera angled at 45° facing the heating mat. One camera was used per crate. Behavior for each litter was filmed continuously for 1 h at the following three timepoints: 24 h before castration (−24 h), immediately post-castration (0 h), and 24 h post-castration (+24 h, Figure 1). Video files were stored on an external hard drive (My Book Duo, Western Digital San Jose, CA).

To accurately assess piglet pain using the UPAPS scale, pigs should be active and awake during the observation period. Therefore, to evaluate activity levels in pigs, a subset of 20, 1-hr videos were randomly selected and observed for piglet activity at −24 and 0 h post-castration time points. Upon observation of these videos, it was determined that piglet activity was greatest within the first 10 min (10.1 ± 4.9 min). Therefore, 4-min video clips were collected using Movavi^©^ Video Suite 22.2.0 (Wildwood, MO, USA) within the first 10-minutes of video recordings for all timepoints. The 4-min video clips were selected based on the methodology proposed by Robles et al., (under review). Video clips were selected as the first 4-minute of the recorded video. If the individual piglet to be observed was not visible or asleep, the video was advanced until the piglet was identified and awake and the 4-minute clip began. A total of 294 videos were clipped and masked by a senior researcher who did not watch the video for behavioral assessment.

### Observer training and UPAPS scoring

One observer (MLS) with over 10 years of experience in the swine industry observed all masked video clips. Prior to data collection, the observer completed two 1-hr training sessions. The training session was conducted by the senior researcher who is a swine veterinarian (MPG) and has more than 10 years of experience evaluating pain behavior. Following the two training sessions, the observer scored 10 masked videos. The order in which the 10 videos were watched was randomized and scores were compared to the senior researcher. An ICC of 0.61 between the observer and senior researcher was required to be met prior to the initiation of data collection.

The pain scale utilized in this study evaluated five behavioral items, with each item sub-categorized into four descriptive levels. A numerical score was designated from “0” to “3”, with a “0” representing normal behavior (free of pain) and “3” corresponding to pronounced behavioral deviation (Table 2). Individual behavioral items and total pain scores were then calculated for each piglet per timepoint.

**Table 2:** The UNESP-Botucatu composite pain scale (UPAPS) for scoring pain in piglets.

Item	Score	Score/criterion	Links to videos
Posture	0	Normal (any position, apparent comfort, relaxed muscles) or sleeping	https://youtu.be/QSosCD2SD4E
	1	Changes posture, with discomfort	https://youtu.be/SpaWsFCrPxE
	2	Changes posture, with discomfort, and protects the affected area	https://youtu.be/VjSlsRrG8yA
	3	Quiet, tense, and back arched	https://youtu.be/pm4hJ5163ao
Interaction and interest in the surroundings	0	Interacts with other animals; interested in the surroundings or sleeping	https://youtu.be/-880STgYq2I
	1	Only interacts if stimulated by other animals; interested in the surroundings.	https://youtu.be/nXjOdwn3dyw
	2	Occasionally moves away from the other animals, but accepts approaches; shows little interest in the surroundings	https://youtu.be/2k2JDr5U6As
	3	Moves or runs away from other animals and does not allow approaches; disinterested in the surroundings	https://youtu.be/se70oYXcWFw
Activity	0	Moves normally or sleeping	https://youtu.be/cC75t7L5-YA
	1	Moves with less frequency	https://youtu.be/lQo9wq8LAn8
	2	Moves constantly, restless	https://youtu.be/YQRJjijLvpk
	3	Reluctant to move or does not move	https://youtu.be/Zyx0G3Wpt8o
Attention to the affected area		A. Elevates pelvic limb or alternates the support of the pelvic limb	https://youtu.be/UD99ftO7HE0
		B. Scratches or rubs the painful area	https://youtu.be/7idfFk1harE
		C. Moves and/or runs away and/or jumps after injury of the affected area	https://youtu.be/u-Pqubom278
		D. Sits with difficulty	https://youtu.be/ETNEOCVV4h0
	0	All the above behaviors are absent	
	1	Presence of one of the above behaviors	
	2	Presence of two of the above behaviors	
	3	Presence of three or all the above behaviors	
Miscellaneous behaviors		A. Wags tail continuously and intensely	https://youtu.be/pU5dGZFNRHc
		B. Bites the bars or objects	https://youtu.be/cF3dsq7gMtk
		C. The head is below the line of the spinal column.	https://youtu.be/ZcIgngclRpI
		D. Presents difficulty in overcoming obstacles (example: another animal)	https://youtu.be/HlvdOI3lGuY
	0	All the above behaviors are absent	
	1	Presence of one of the above behaviors	
	2	Presence of two of the above behaviors	
	3	Presence of three or all the above behaviors	

### Rescue analgesia

Following video scoring for each treatment, the observer was required, based on experience, to mark whether the piglet required (yes) or did not require (no) analgesic intervention due to breakthrough pain. This is most commonly referred to rescue analgesia in the literature and was conducted for each video clip. Total counts were calculated for piglets requiring or not requiring rescue analgesia by treatment and timepoint.

### Statistical analysis

A multivariable model was built at the piglet level for the average of total pain scores (outcome of interest). The averages of total pain scores were analyzed as repeated measures by an analysis of variance applying a multilevel model, and the structure of covariance was chosen according to the Bayesian information criterion. Treatment and timepoint were included as the fixed effects. Piglet was included as a random effect to account for repeated measurements on individual animals. Statistical significance was declared at *P* ≤ 0.05 and a tendency was declared at 0.05 < *P* ≤ 0.10. All data were analyzed using SAS version 9.4 (28).

For the rescue analgesia data, test of homogeneity (Chi square $X^{2}$) was used to determine if the distribution of the piglets in pain requiring rescue analgesia was the same among all treatments (C, CF, S, SF).

## Results

Data was collected on a total of 98 piglets. Average piglet age and body weight was 2.5 days (±0.9-day SD) and 2.2 kg (±0.5 kg SD) respectively. Average parity of sows whose piglets were enrolled on the study was 3.1 (±1.4 SD; Table 3).

**Table 3:** Mean ± SD. Descriptive statistics per variable at enrollment for C, CF, S and SF groups. Tukey's Studentized Range (HSD) with α = 0.05.

	C	CF	S	SF	Overall mean	*P*-value
Parity	3.3 ± 1.4	2.8 ± 1.5	3.2 ± 1.4	3.3 ± 1.3	3.1 ± 1.4	0.44
Total born	15.0 ± 4.3	13.4 ± 3.5	14.6 ± 3.7	16.0 ± 3.5	14.75 ± 3.8	0.08
Liveborn	13.8 ± 3.8	12.6 ± 3.4	13.7 ± 3.3	14.9 ± 3.2	13.71 ± 3.5	0.11
Stillborn	0.8 ± 0.9	0.5 ± 0.7	0.6 ± 0.8	0.8 ± 0.7	0.66 ± 0.76	0.46
Mummies	0.4 ± 0.7	0.4 ± 0.7	0.4 ± 0.7	0.4 ± 0.7	0.4 ± 0.7	0.99
Age (days)	2.6 ± 1.4	2.3 ± 0.7	2.3 ± 0.7	2.6 ± 0.7	2.5 ± 0.9	0.43
Weight	2.1 ± 0.5	2.2 ± 0.5	2.2 ± 0.5	2.1 ± 0.6	2.2 ± 0.5	0.97

### Effect of the drug, procedure and timepoint on total pain scores

**Figure 2:**
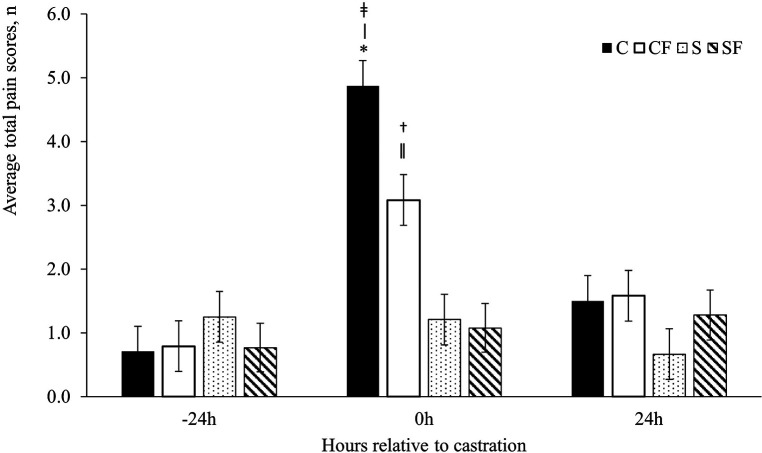
Mean ± SEM total pain scores for piglets in the C, CF, S and SF groups. Timepoint (*P* < 0.0001), treatment (*P* < 0.001) and treatment by timepoint (*P* < 0.0001) effect. Pairwise comparisons with *P* ≤ 0.03: ǂ C vs. SF; ǀ C vs. S; † CF vs. SF; ǁ CF vs. S. Pairwise comparisons with *P* ≤ 0.05: * CF vs. C.

The UPAPS effectively distinguished varying levels of painful and non-painful states in castrated and non-castrated piglets. There was a timepoint (*P* < 0.0001), treatment (*P* < 0.001) and treatment by timepoint (*P* < 0.0001) effect on total pain score (Figure 2).

Total average pain scores did not differ at −24 h (*P* > 0.05) or at 24 h post-castration (*P* > 0.05) between treatments or timepoint (*P* > 0.05). However, immediately post-castration (0 h), piglets in the C and CF group had higher total average pain scores than piglets in the S (*P* < 0.03) and SF (*P* < 0.01) groups (Table 4). Piglets in the CF group demonstrated lower pain scores at 0 h compared to C piglets (*P* < 0.05).

**Table 4:** Descriptive statistics for total UPAPS scores for treatment groups^a^ across all timepoints^b^.

	Statistics	Mean	SD	Median	Min	Max
	**Timepoint**	**−24 h**				
Treatments	C	0.7	1.0	0.5	0	4
	CF	0.8	0.8	1.0	0	3
	S	1.3	1.2	1.0	0	4
	SF	0.8	0.7	1.0	0	2
	Statistics	Mean	SD	Median	Min	Max
	**Timepoints**	**0 h**				
Treatments	C	4.9	3.7	4.0	0	12
	CF	3.1	3.8	1.0	0	11
	S	1.2	0.9	1.0	0	3
	SF	1.1	0.6	1.0	0	2
	Statistics	Mean	SD	Median	Min	Max
	**Timepoints**	**24 h**				
Treatments	C	1.5	0.9	1.5	0	3
	CF	1.6	2.0	1.0	0	10
	S	0.7	0.6	1.0	0	2
	SF	1.3	2.2	1.0	0	10

^a^ Timepoints: 24 h before castration (−24 h), immediately post-castration (0 h), and 24 h post castration (+24 h post-castration)

^b^ Treatments: CF, Transdermal flunixin (TDF) applied topically followed by surgical castration (3.33 mg/kg; *n *= 24), SF, TDF applied topically followed by sham castration (3.33 mg/kg; *n* = 26), S, physiological saline followed by sham castration (*n *= 24), and C, physiological saline followed by surgical castration (*n *= 24).

### Rescue analgesia

**Figure 3:**
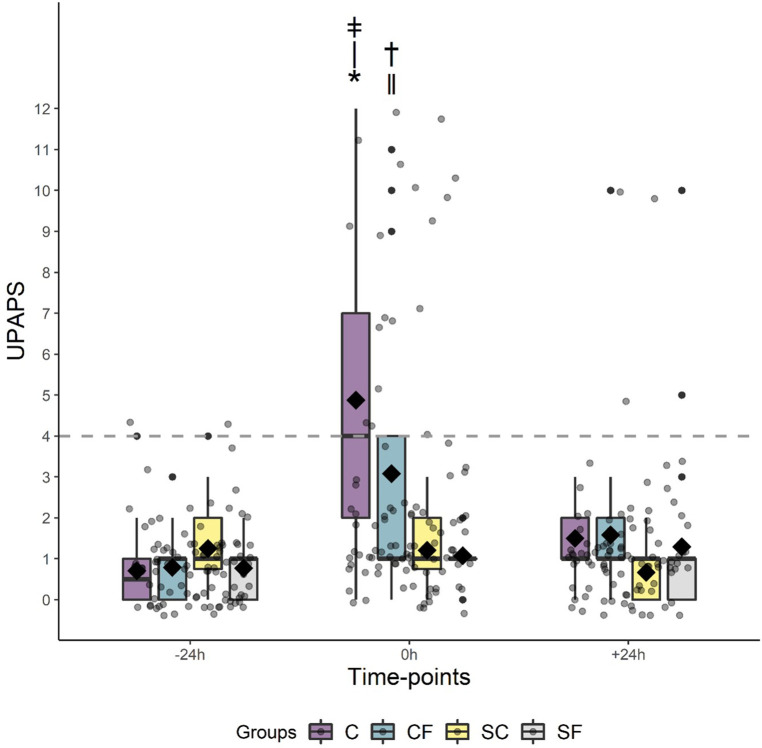
Boxplot for rescue analgesia for piglets in the C, CF, S and SF groups per timepoints at −24 h, 0 h and 24 h using a cutoff = 4. Chi square $X^{2}$ with *P* ≤ 0.05: * ǂC vs. †CF.

When assessing rescue analgesia (i.e., piglets identified by the observer as requiring analgesic intervention), rescue analgesia was needed the most in C piglets (Chi square $X^{2}$, *P *<0.0001), with 54% of C pigs requiring rescue analgesia compared to 29%, 8% and 0% for CF, SF and S piglets respectively (Figure 3).

## Discussion

Around the world most male piglets are castrated (1) and, for most part, this painful procedure is conducted without analgesia or anesthesia (29). Currently, there are no drugs specifically labeled to control pain in swine and the US industry is actively working on obtaining an FDA approved product to be used in conjunction with painful husbandry procedures such as castration (30). Transdermal flunixin was effective in mitigating pain in livestock species such as dairy cattle (31) and goats (32). This product shows promise as a realistic option to be implemented for pain management on large-scale farm systems. Therefore, the objective of this study was to evaluate the efficacy of TDF in mitigating castration pain utilizing a previously validated piglet acute pain scale (UPAPS).

Results from this study demonstrated that the UPAPS effectively distinguished varying levels of pain states in castrated piglets as observed by deviations in total pain scores across timepoints and among groups. These findings coincide with work conducted in other species such as sheep (17), cattle (16), and donkeys (19), as well as pre-weaned (20) and post-weaned pigs (15). The UPAPS is unidimensional in nature with an excellent internal consistency (Cronbach's alpha ≥ 0.85) and inter-observer agreement (ICC = 0.81). The UPAPS has repeatedly been shown to distinguish between painful and non-painful states in pigs with excellent sensitivity (92.9%) and moderate specificity (78.6%, 20). The UPAPS is a sensitive tool that not only detects pain states but can also be used to validate pharmaceutical products for pain mitigation. Given these promising results, future work should focus on the wide-scale implementation of the scale to be used by swine caretakers, producers, and veterinarians on farm.

In this study, administering TDF 24 h prior to castration mitigated pain immediately post-castration to the majority of piglets enrolled on the trial as demonstrated by decreased average total pain scores in CF piglets compared to C piglets and decreased identification of CF piglets requiring analgesic intervention to control breakthrough pain compared to C piglets. Flunixin meglumine is a common NSAID used on swine farms to control fever associated with respiratory disease and has been proven effective in mitigating lameness pain in swine (23). This drug has the potential to mitigate both acute and chronic pain in swine and can be used in an extra label manner under the discretion of a veterinarian (33). Banamine® Transdermal has even more potential to be used on swine farms given it is one of the most realistic pain management options to implement given the ease of applicability and long half-life. Topical drugs such as TDF work by crossing the stratum corneum skin layer and diffusing slowly through intercellular lipids reaching maximum concentration around 24 h post-administration (34). Previous pharmacokinetic work assessing TDF (36) reported low bioavailability in in pre-weaned piglets (7.8%), compared to PO and IM (>99%,). However, bioavailability is not equivalent to efficacy given TDF's unique mode of action and local efficacy, systemic absorption represented as bioavailability may not be critical. Considering these unique pharmacokinetic properties, TDF was administered 24 h prior to castration to maximize drug concentration. This delayed absorption is a distinct advantage for the swine industry, given producers can apply the drug topically the day before castration, thus maximizing drug concentration at the time of castration. However, labor efforts should be considered given pigs will need to be handled twice, although previous work suggests extra cost is minimal for large production systems (35).

Specific to castration, previous work conducted in lambs (23), goats (24) and calves (25) has demonstrated flunixin meglumine efficacy in controlling procedural pain utilizing both behavior and physiological measurements. To the author's knowledge, there is only one publication assessing the efficacy of TDF in controlling castration pain specific to piglets (28). Merenda and colleagues (2022) assessed the efficacy of TDF administration on serum prostaglandin E_2_ (PGE_2_) and cortisol concentrations for piglets undergoing castration using the same treatment groups described in this study. Serum PGE_2_ concentrations for piglets in the CF and C group were not different, regardless of the timepoint. CF treated piglets tended to demonstrate lower cortisol concentrations immediately post-castration compared to C piglets and suggests that TDF is demonstrating some mitigating effects on stress associated with castration. Future work pairing physiological parameters in conjunction with behavioral assessment like UPAPS, may provide a clearer image on the role TDF has on mitigating castration pain.

Total average pain scores at 24 h were not different in castrated piglets when compared to scores at −24 h. This is contrast with work that demonstrates pain sensitivity beyond 24 h (35) and may be due to the fact that the pain scale cannot effectively identify mild and/or chronic pain response. At this time, the UPAPS scale has only been used up to 24-hour post-castration in piglets, therefore, the scale is currently optimal for the evaluation of perioperative pain and future work should evaluate the efficacy of the scale for chronic pain assessment given castration pain can last up to 5–7 days post-castration. Future work should focus on additional timepoints post-castration to determine varying pain levels and utilize alternative pain models quantify and validate the scale's efficacy for different pain modalities.

Finally, 8% of piglets in the SF group were observed to require drug relief. This is possible due to that piglet might have fallen within the diagnostic uncertain zone with a score of 4–5, yielding false positive results, like observed in the original study that validated UPAPS (15), where, according to different observers, 7% of pigs were in the diagnostic uncertain zone at the baseline preoperative timepoint. Additionally, pain-related behaviors used to diagnose pain are not pathognomonic of pain, these behaviors may occur due to other phenomena than pain. And this could be a limitation of using behaviors to assess pain, although this happens in very few cases.

## Animal welfare implications and conclusions

The UPAPS is a sensitive and effective tool for objectively assessing castration pain in piglets and the validation and use of this scale will have significant impacts in the approval and availability of pharmaceutical productions to mitigate pain in swine. Administering TDF 24 h prior to castration demonstrated a decrease in total pain scores and decreased the percentage of pigs requiring analgesic intervention to control breakthrough pain when compared to castrated piglets receiving no pain mitigation (CF piglets 29% vs. C piglets 54%). This drug shows significant promise as a realistic tool to be implemented on commercial swine farms, given is ease of applicability and long half-life. Total average pain scores at 24 h were not different in castrated piglets when compared to scores at −24 h and future work should focus on utilizing alternative measurements to assess chronic pain in swine utilizing different pain models.

## Data Availability

The raw data supporting the conclusions of this article will be made available by the authors, without undue reservation.
